# A randomized control trial to compare mortality in recipients of leucoreduced and non-leucoreduced whole blood transfusion in patients with cancer in Uganda

**DOI:** 10.1186/s12885-024-12445-w

**Published:** 2024-06-03

**Authors:** Clement D. Okello, Jackson Orem, Martin Nabwana, Noah Kiwanuka, Andrew W. Shih, Nancy Heddle, Harriet Mayanja-Kizza

**Affiliations:** 1 Uganda Cancer Institute, Kampala, Uganda; 2 Makerere University, Johns Hopkins University Research Collaboration, Kampala, Uganda; 3 School of Public Health, College of Health Sciences, Makerere University, Kampala, Uganda; 4 Department of Pathology and Laboratory Medicine, University of British Columbia, Vancouver, BC Canada; 5 Michael DeGroote Centre for Transfusion Research, Department of Medicine, McMaster University, Hamilton, ON Canada; 6 Department of Medicine, College of Health Sciences, Makerere University, Kampala, Uganda

**Keywords:** Mortality, Leucoreduced whole blood, Uganda

## Abstract

**Background:**

Mortality benefit of transfusion with leucoreduced whole blood has not been demonstrated in the sub-Saharan Africa (SSA). We compared mortality in patients with cancer transfused with leucoreduced and non-leucoreduced whole blood in a SSA setting.

**Methods:**

An open-label randomized controlled trial was conducted at the Uganda Cancer Institute where participants were randomized in a 1:1 ratio into the leucoreduced and non-leucoreduced whole blood transfusion arms. Leucocyte filtration of whole blood was performed within 72 h of blood collection. Patients aged ≥ 15 years who were prescribed blood transfusion by the primary physicians were eligible for study enrolment. Mortality difference was analyzed using intention-to-treat survival analysis and cox proportional hazard model was used to analyze factors associated with mortality.

**Results:**

There were 137 participants randomized to the leucoreduced and 140 to the non-leucoreduced arms. Baseline characteristics were similar between the two arms. The median number of blood transfusions received was 1 (IQR, 1–3) unit and 2 (IQR, 1–3) units in the leucoreduced and non-leucoreduced arms respectively, *p* = 0.07. The 30-day mortality rate in the leucoreduced arm was 4.6% (95% CI, 2.1–10) and was 6.2% (95% CI, 3.2–12.1) in the non-leucoreduced arm (*p* = 0.57), representing an absolute effect size of only 1.6%. Increasing age (HR = 0.92, 95% CI, 0.86–0.98, *p* = 0.02) and Eastern Co-operative Oncology Group (ECOG) performance score of 1 (HR = 0.03, 95% CI, 0.00–0.31, *p* < 0.01) were associated with reduced 30-day mortality.

**Conclusions:**

The study failed to demonstrate mortality difference between cancer patients transfused with leucoreduced and non-leucoreduced whole blood. Although this study does not support nor refute universal leucoreduction to reduce mortality in patients with cancer in SSA, it demonstrates the feasibility of doing transfusion RCTs in Uganda, where a multi-center trial with an appropriate sample size is needed.

**Trial registration:**

Pan African Clinical Trial Registry, https://pactr.samrc.ac.za/ (PACTR202302787440132). Registered on 06/02/2023.

## Background

Blood transfusion is a life-saving intervention, but safety concerns exist especially in low- and middle-income countries [1]. Many safety concerns associated with blood transfusion have been linked to the presence of donor leucocytes in the blood product transfused. Though whole blood transfusions are increasingly common in resuscitative settings, blood transfusion practices in most centres in sub-Sahara Africa (SSA) are mainly with whole blood that contains leucocytes. This practice is not shared by many developed nations that now increasingly transfuse patients with specific blood components including red blood cells and also routinely use blood products that are pre-storage leucoreduced.

Transfusion with leucoreduced blood has been shown to significantly reduce some complications associated with blood transfusion such as febrile non-haemolytic transfusion reaction (FNHTR) [2–4], platelet refractoriness [5–7], and infection [8–10]. A retrospective multicenter Canadian study found association with decreased mortality after the introduction of universal leucoreduction in their patient cohorts, which included those undergoing cardiac surgery, hip fracture repair and intensive care unit admission [11]. The aforementioned studies demonstrate the benefits of leucoreduced blood transfusion in high income countries, where whole blood is primarily used in critically ill patients with substantial blood loss and where there is relatively adequate blood supply [12]; hence the results are not generalizable to SSA.

On the contrary, the overwhelming requirement for blood transfusion in SSA coupled with limited blood supply implies that most blood units are transfused as soon as they are collected; meaning that, relatively fresh blood units are commonly used for transfusion in these settings. There is increasing evidence to suggest that transfusion with fresh blood may be harmful [13–15]. Although transfusion with leucoreduced blood has been associated with some beneficial outcomes [2–10], these evidence have not been demonstrated in SSA. Recently, we reported the 30-day mortality rate in transfused patients in a tertiary hospital in Uganda to be 25.2%, where cancer was a top indication for transfusion [16]. Cancer was also the top indication for transfusion in an earlier study in Uganda [17]. In the current study, we sought to explore the benefits of transfusion with leucoreduced blood in patients with cancer. Thus, the primary objective of the current study was to compare mortality rates in participants transfused with leucoreduced and non-leucoreduced whole blood; and the secondary exploratory objectives were to determine the factors associated with mortality and to compare acute transfusion reactions at the Uganda Cancer Institute.

## Methods

### Study design

An open-label randomized controlled trial (RCT) was performed where participants were randomized using permuted block randomization. Block sizes of 6 were used with a 1:1 allocation ratio across the leucoreduced and non-leucoreduced whole blood transfusion arms. Study randomization numbers were generated using a computer-based random number generation sequence. The randomization numbers were then used to assign participants into the leucoreduced and non-leucoreduced whole blood transfusion arms.

### Study setting

The study was conducted at the Uganda Cancer Institute (UCI), a tertiary cancer treatment facility located in Kampala, Uganda. Patients with various types of cancers including solid tumours, and haematological cancers are treated at the UCI. Blood transfusion at the UCI is prescribed by a physician, and follows the prescription guidelines of the Ugandan Ministry of Health which recommends blood transfusion based on the clinical condition of the patient, and especially when the haemoglobin level is below 7 g/dL, or below 6 g /dL for patients with sickle cell anaemia [18].

Leucoreduced and non leucoreduced whole blood were prepared and provided by the Uganda Blood Transfusion Services (UBTS), Nakasero-Kampala. Prior to hospital deliveries, all blood products were serologically tested and released only when found negative for human immunodeficiency virus, hepatitis B and C, and syphilis. Non-leucoreduced whole blood transfusion was the current standard of care in Uganda at the time of this study. Whole blood units provided by UBTS for transfusion were preserved in citrate phosphate dextrose adenine (CPDA-1) and kept under refrigerated storage at 1 to 6 °C. Leucoreduction was performed within 72 h of blood collection at the UBTS for the purpose of this study only using commercially acquired equipment (LEUCOLAB LCG4b, Macopharma-Rue Lorthiosis, Mouvaux-France) in accordance with the product manual. The leucoreduced whole blood units were labeled for easy identification. At the hospital, pre-transfusion testing consisted of recipient’s ABO and Rhesus D blood typing, and a room temperature immediate spin cross-match – all performed using the tile method. Transfusions with non-study blood products including platelets were allowed if indicated as standard of care.

### Study procedures

This study was executed by the participation of a multidisciplinary team. The study staff comprised of the laboratory technologists from the UBTS and the UCI, nurses (research assistants), study coordinators, statistician and the PI. The laboratory technologists at the UBTS processed the blood products as per their normal standard of operations. Additionally, they randomly picked the processed whole blood units for leucoreduction using the study standard operating procedures (SOPs). On average, about 7–10 blood units were transfused in the study per week. Leucocyte filters and reagents for RBC allo-antibody testing were procured locally in Kampala. Although these products were manufactured in Europe, they were utilized as per the manufacturer’s protocols and none expired during the study.

The study nurses, coordinators, hospital laboratory technologist and the principal investigator were all based at the Uganda Cancer Institute, while the statistician was based in another institution, but within Kampala. Prior to study engagements, all the study staff were required to have undergone professional training in their respective fields; and in addition, they were trained on their study roles and Good Clinical Practice (GCP) and Good Clinical Laboratory Practice (GCLP) where appropriate. All study activities were guided by the SOPs to ensure consistency. The SOPs were written by the study team with guidance from the product manuals and to acceptable standards. Study meetings were regularly held by the study staff. The study coordinators were responsible for coordinating all the study activities, while the study PI provided oversight for the study. The study monitor and members of the Data Safety and Monitoring Board (DSMB) were independent of the study.

Potential participants were identified by the hospital laboratory technologist based on the submitted request for blood transfusion and the eligible participants were consecutively recruited by the study nurses until the desired sample size was obtained.

### Study participants

Participants were eligible for the study if they met the following inclusion criteria: ≥ 15 years of age; likely to need blood transfusion as judged by the primary care physician; and, admitted to the Uganda Cancer Institute. Eligible patients were excluded based on the following criteria: required urgent blood transfusion because study procedures would delay transfusion; participating in a competing study; received transfusion within the previous 48 h prior to study enrollment; compatible blood units at the time of enrollment were not available; were previously enrolled in the same study; had no pre-transfusion blood sample for testing; and, had altered mental status. Recruitment was based on provision of informed consent or assent.

### Data Collection

All data were manually recorded on a standardized data collection form. Data collected included: demographic information; diagnoses; baseline performance score based on the Eastern Cooperative Oncology Group (ECOG) criteria; history of previous pregnancies or parity; number of previous blood transfusion and transfusion received during the study; and, ABO and Rhesus blood group. Transfusion with non-study blood products were captured. Data collected were verified for completeness and accuracy by the principal investigator. All patients’ information was anonymized after data verification. Verified data were coded, and entered into a database using Epidata version 3.1 (Epidata association, Denmark) and cleaned to ensure accuracy before exporting to STATA Version 15 (StataCorp, USA) for analysis. Data were locked in a secure place and the database was kept in a computer secured with a password. Study approvals were obtained from the Makerere University School of Medicine Research Ethics Committee (Ref. 2017 − 106) and the Uganda National Council for Science and Technology (Ref. HS 2705). The RCT was registered at the Uganda National Drug Authority (CTA-0137) and the Pan African Clinical Trial Registry (https://pactr.samrc.ac.za; PACTR202302787440132. Registered on 06/02/2023).

### Study outcome

The primary outcome was all-cause mortality within 30 days of study enrollment. The secondary outcomes were any adverse reactions associated with blood transfusion, which included: febrile non-haemolytic transfusion reaction (FNHTR), transfusion associated lung injury (TRALI), transfusion transmitted infection (TTI), acute haemolytic transfusion reaction (AHTR), transfusion associated circulatory overload (TACO), urticarial/anaphylaxis reactions, and transfusion associated graft versus host disease (TA-GVHD); and factors associated with 30-day all-cause mortality. Adjudication of adverse reactions was done by the study participant’s primary physicians. After discharge from hospital, participants were requested to report any adverse events to the primary physician or study team for appropriate treatment.

### Statistical analysis

We estimated that with enrolment of 262 participants the study would have 90% power to detect a significant difference at 5% level, assuming a 30-day all-cause mortality of 25.2% in recipients of non-leucoreduced blood [16] and 10.2% (absolute effect size of 15%) in recipients of leucoreduced blood [19]. Demographic and clinical characteristics were described using frequencies and percentages. Continuous data were described using mean and standard deviation for parametric data and median and interquartile range (IQR) for nonparametric data. Unadjusted comparisons of percentages were made with Fisher’s exact test. The Wilcoxon rank sum test was used for comparisons of continuous outcomes with skewed distribution. The Kaplan-Meier method was used for censored outcomes.

The two transfusion arms were compared using the intention-to-treat survival analysis. The primary analysis (rates of mortality) were determined by dividing the number of deaths by the person-years of follow-up and the comparison was based on a log-rank test of the difference between the two transfusion arms in the time-to-death, with no adjustments for baseline covariates. Participants lost to follow up were included in the analysis and were censored on the last recorded date of review. Hazard ratios and 95% confidence intervals were generated.

A Cox proportional-hazards model was used to adjust for differences in participants’ baseline characteristics. At bivariable analysis, all the independent variables were regressed against 30 – day mortality and only variables with p-value < 0.1 and diagnosis were considered for multivariable analysis.

### Data safety and monitoring board

An independent Data Safety and Monitoring Board (DSMB) was composed of a hematologist, a study methodologist and a biostatistician, all of whom were not affiliated to the study. A pre-specified interim analyses were set at information fractions (IF) of 50% and 75% corresponding to post randomization follow up periods. The Lan – DeMets O’Brien – Fleming alpha spending boundaries for rejecting the null hypothesis at each data look were used with futility – efficacy boundaries of 0.33–2.96 and 1.29–2.36 corresponding to 50% IF and 75% IF respectively. If the value of the observed Z statistics for comparing the two groups exceeded the boundaries at the stated IF, in absolute terms, the null hypothesis of no difference between the study groups were rejected. The O’Brien Fleming alpha spending function was chosen because it avoided early termination of the study owing to the boundaries being very conservative early in the study but less conservative as information increases. Statistical significance for all analyses was set at *p* < 0.05 (two-sided).

### Quality of whole blood leucoreduction

To check for the adequacy of leucoreduction, the initial 137 units of leucoreduced whole blood transfused at the time of enrolment were sampled as a measure of quality control. Of these, 60 (43.8%) units, 54 (39.4%) units and 23 (16.8%) units were leucoreduced within 24 h, 48 h and 72 h of collection respectively. Based on the review by Sharma et al., 72 h is an acceptable timeframe within which pre-storage leucoreduction can be performed [20]. All the leucoreduced blood units contained < 1 × 10^6^ leucocytes per unit after enumeration using the Sysmex XN*-*1000 CBC machine.

## Results

### Participants randomized

**Fig. 1:**
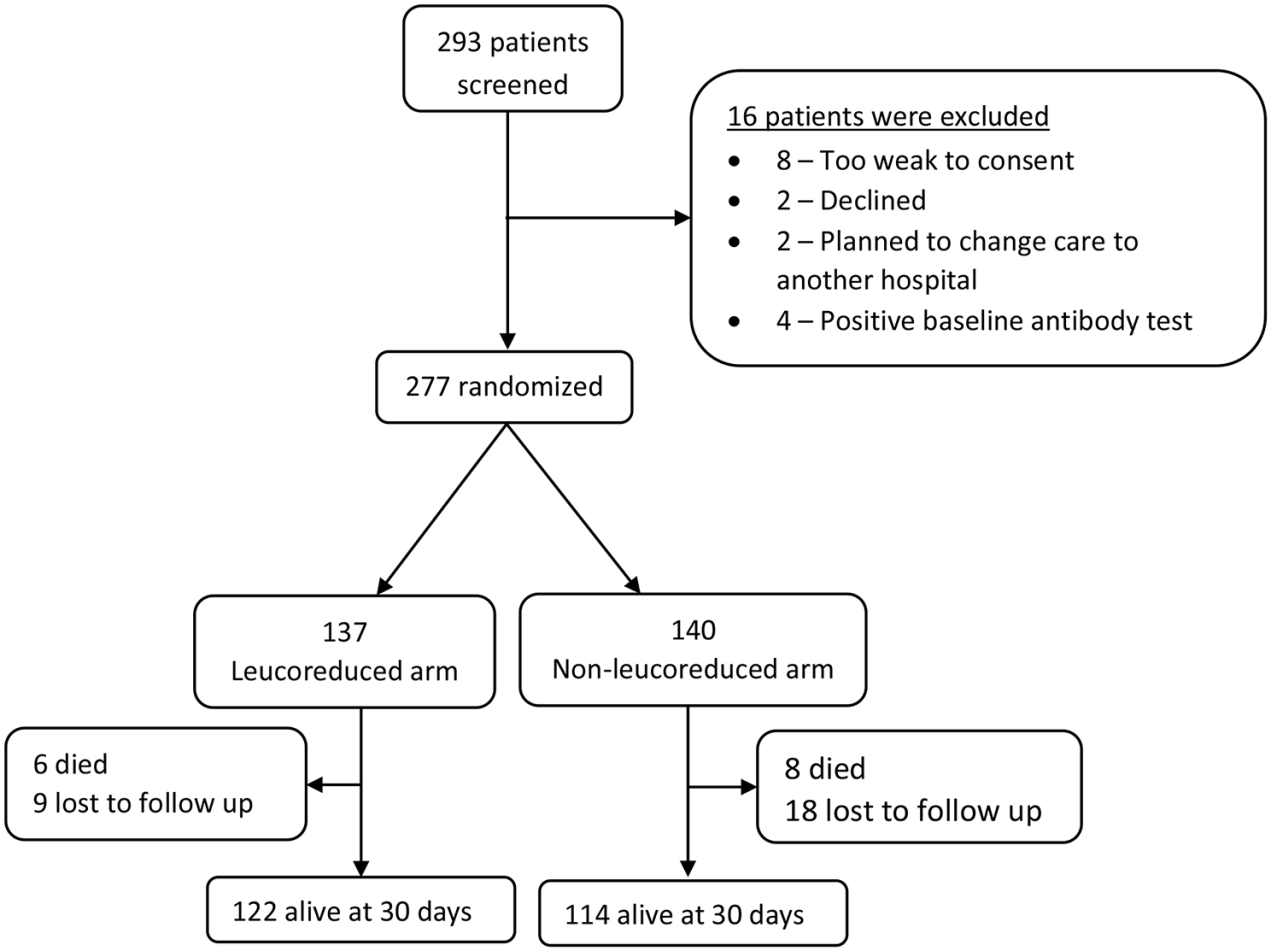
Trial profile: Screening, randomization & follow up

Two hundred ninety three patients were screened over a 12 month period from 02 December 2020 to 30 November 2021, of whom 277 participants were randomized to receive either leucoreduced whole blood transfusion (*n* = 137) or non-leucoreduced whole blood transfusion (*n* = 140) and were analyzed; a total of 16 of the screened patients were excluded based on the eligibility criteria, including four participants who had positive antibody tests at baseline, Fig. 1.

### Baseline characteristics

Baseline characteristics of the participants at the time of randomization including age, ECOG physical performance status, number of prior pregnancies, ABO and Rhesus blood types, prior exposures to blood transfusions (Table 1) and diagnoses (Table 2) were similar for both study arms. The three most common diagnoses were gynaecological cancers (*n* = 88, 31.8%), acute leukaemia (*n* = 35, 12.6%), and gastrointestinal cancers (*n* = 25, 9.0%), Table 2. Twenty-six (9.4%) participants had concomitant HIV infection, of whom 15 (5.4%) had cervical cancer, 05 (1.8%) Kaposi sarcoma, 2 (0.7%) NHL, 2 (0.7%) vulvar cancer, 1 (0.4%) oesophageal cancer and 1 (0.4%) breast cancer.

**Table 1:** Baseline characteristics of randomized participants

Characteristics	Overall (*N* = 277)	Leucoreduced arm (*n* = 137)	Non-leucoreduced arm (*n* = 140)
**Median age (IQR), Years**	40 (30, 52)	42 (33, 55)	39 (26.5, 50.0)
**Female gender, n(%)**	168 (60.7)	91 (66.42)	77 (55)
**ECOG performance score, n(%)**			
1	63 (22.7)	26 (19.0)	37 (26.4)
2	152 (54.9)	84 (61.3)	68 (48.6)
3	51 (18.4)	23 (16.8)	28 (20)
4	11 (4.0)	4 (2.9)	7 (5)
**Parity, Mean (SD)**	4 (2.8)	4 (2.7)	5 (2.8)
**Pre-transfusion Hb, Mean (SD)**	6.3 (1.1)	6.4 (1.0)	6.1 (1.2)
**ABO blood group, n(%)**			
A	63 (22.7)	28 (20.4)	35 (25)
B	57 (20.5)	35 (25.5)	22 (15.7)
AB	13 (4.7)	3 (2.2)	10 (7.1)
O	144 (52.0)	71 (51.8)	73 (52.1)
**Rhesus positive blood group, n(%)**	274 (98.9)	136 (99.3)	138 (98.6)
**Previous transfusion, Median (IQR)**			
Whole blood, units	2 (1–12)	2 (1–11)	2 (1–11)
Packed cells, units	2 (1–11)	2 (1–6)	2 (1–11)
Platelets, units	8 (2–11)	5 (5–5)	11 (5–11)

Note: All data are expressed as number and % unless otherwise stated. All blood products transfused prior to enrolment were non-leucoreduced. ECOG – Eastern Cooperative Oncology Group; Hb – Haemogloblin; SD – Standard deviation and IQR -interquartile range

**Table 2:** Participant diagnosis

Diagnosis	Overall (*N* = 277)	Leucoreduced arm (*n* = 137)	Non-leucoreduced arm (*n* = 140)
Gynaecological Cancers	88 (31.8)	49 (35.8)	39 (27.9)
Acute Leukemia	35 (12.6)	17 (12.4)	18 (12.9)
GastrointestinalCancers	25 (9.0)	12 (8.8)	13 (9.3)
Urological Cancers	22 (7.9)	10 (7.3)	12 (8.6)
Breast Cancer	20 (7.2)	13 (9.5)	7 (5)
Skin and Sarcomas	19 (6.9)	8 (5.8)	11 (7.9)
Lymphoma	16 (5.8)	5 (3.6)	11 (7.9)
Non-Cancers	9 (3.3)	3 (2.2)	6 (4.3)
Chronic Leukemias	9 (3.3)	3 (2.2)	6 (4.3)
Kaposi Sarcoma	8 (2.8)	4 (2.9)	4 (2.9)
Multiple Myeloma	7 (2.6)	6 (4.4)	1 (0.7)
Head and Neck	6 (2.2)	3 (2.2)	3 (2.1)
Brain tumors	4 (1.5)	1 (0.7)	3 (2.1)
Bone Tumors	3 (1.1)	0	3 (2.1)
Other Cancers	6 (2.2)	3 (2.2)	3 (2.1)

### Transfusions during the study

The median number of blood units received during the 30 – day study period was 1 (IQR, 1–3) unit and 2 (IQR, 1–3) units in the leucoreduced and non-leucoreduced arms respectively, *p* = 0.07. Four participants in the leucoreduced arm also received non-leucoreduced blood (analyzed by intention to treat). Only two (0.7%) participants received ≥ 10 units of blood transfusion during the study period, all in the non-leucoreduced arm. Platelets transfusion happened in four participants in the leucoreduced arm and 5 participants in the non-leucoreduced arm; of these, three of the four participants in the leucoreduced arm and all the five participants in the non-leucoreduced arm were transfused with ≥ 10 units of RhD-matched platelets during the study. No participant was transfused with packed red cells.

### Mortality rates and factors associated with mortality

**Fig. 2:**
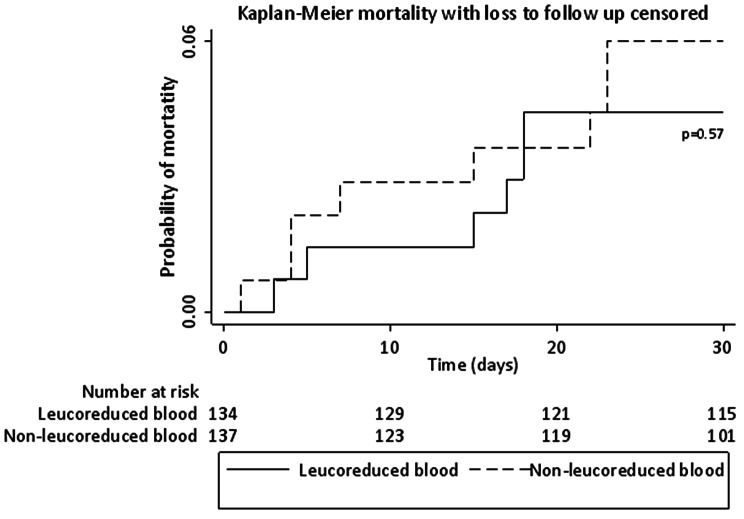
Kaplan-Meier mortality estimates

**Fig. 3:**
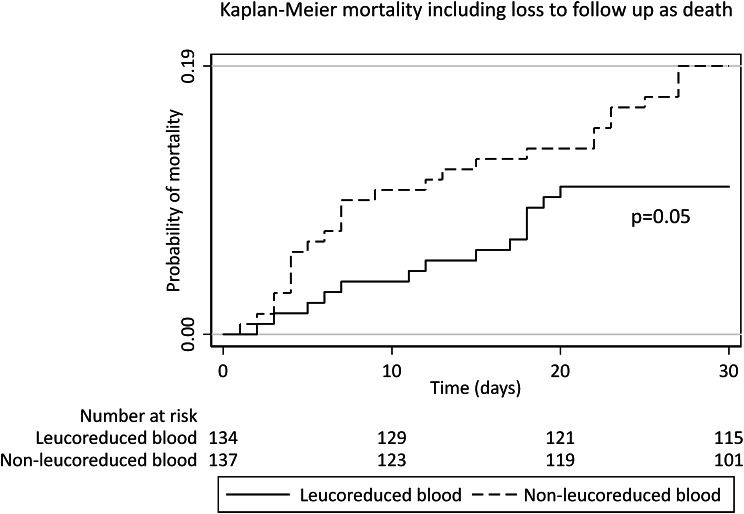
Kaplan-Meier mortality graph including attribution of loss to follow up as participant death (worst case scenario)

The 30-day mortality rate in the leucoreduced arm was 4.6% (95% CI, 2.1–10) and was 6.2% (95% CI, 3.2–12.1) in the non-leucoreduced arm, *p* = 0.57, Fig. 2. The overall loss to follow-up was 11.9% including nine participants in the leucoreduced arm and 18 participants in the non-leucoreduced arm. However, if the reason for participant lost to follow up was attributed to death (worst case scenario), the 30-day mortality rate still did not differ between the two transfusion arms, that is, 10.5% (95% CI, 6.3–17) in the leucoreduced arm and 19% (95% CI, 13.3–26.6) in the non-leucoreduced arm, *p* = 0.05, Fig. 3. Increasing age (HR = 0.92, 95% CI, 0.86–0.98, *p* = 0.02) and ECOG performance score of one, that is, being less ill (HR = 0.03, 95% CI, 0.00–0.31, *p* < 0.01) were associated with reduced 30-day mortality at both bivariable and multivariable analyses.

### Other adverse acute transfusion reactions

One (1/137, 0.01%) participant in the leucoreduced arm developed febrile non-haemolytic transfusion reaction; and one (1/140, 0.01%) participant in the non-leucoreduced arm developed urticaria, *p* = 0.98. There were no other adverse transfusion reactions reported.

### Trial continuation despite futility review

The data and safety monitoring board evaluation at 50% point of information fraction did not cross either efficacy or futility boundaries. Evaluation at 75% point of information fraction crossed the futility boundary. However, the study was allowed to complete recruitment because there was no added harm attributed to the use of leucoreduced blood in the study, the study was recruiting quickly, and the leucocyte filters were readily available for the study (and would have expired and been discarded if not used for the study).

## Discussion

This study failed to demonstrate any mortality difference between patients with cancer transfused with leucoreduced whole blood or non-leucoreduced whole blood in a sub-Saharan African setting. However, the study was likely underpowered to have a definitive conclusion. Results of the secondary exploratory outcomes also showed no differences in the occurrence of acute blood transfusion reactions between the two study arms; and increasing age among the study participants and participants who were less ill had significantly reduced mortality rate.

These findings may be consistent with the results of a study by Dzik et al. in North America, where 1,355 participants were randomized to leucoreduced blood component transfusion and 1,425 participants to non-leucoreduced blood component transfusion, and where no differences in mortality were found [21]. Our results may extend this observation to the population of patients with cancer and whole blood, as opposed to a mix of both surgical and general medical patients in the study by Dzik et al. [21], but further study is needed to confirm. We did not find any comparative study in the SSA setting.

Though our study is inconclusive, we hypothesized leucoreduced whole blood may improve patient outcomes as the mortality benefits of transfusion with leucoreduced blood components have been reported in other studies, especially those involving surgical patients. A randomized controlled trial conducted in European patients undergoing cardiac surgery showed increased mortality in patients who received non-leucoreduced blood compared to leucoreduced blood. The increased mortality in the non-leucoreduced blood recipients was attributed to the higher incidence of postoperative infections [22]. There was also reduced mortality following transfusion with leucoreduced blood in a retrospective before –and – after implementation study in a high-risk surgical cohort comprising of 14,786 patients across 23 hospitals in Canada over a two-year period [23]. It is postulated that the adverse effects on non-leucoreduced blood transfusion selectively seen across the surgical studies [22, 24–27] may be associated with factors unique to surgery including exposure to the extracorporeal circuit, hypothermia, and reperfusion injury [28] that might be mitigated by leucoreduction. Our study instead assessed cancer patients.

Based on exploratory analyses using univariable and multivariable strategies, we noted increasing age and being less ill (based on lower ECOG performance scores) in transfused participants associated with reduced mortality. We also found no differences in acute transfusion reactions in the study participants. However, due to inadequate statistical power in the current study, we contend that our data are unable to support any conclusions here.

The strength of this study is the demonstration of the feasibility of performing transfusion RCTs in SSA. The number of patients screened in our study was consistent with previous reports showing high demands for transfusion in patients with cancer in Uganda [17]. The study was undertaken through the representation of a multidisciplinary steering committee with regular meetings to ensure study protocols were followed with ongoing troubleshooting. The trial protocol was also published in the Pan African Clinical Trial Registry, a public trials registry (https://pactr.samrc.ac.za/) developed to ensure transparency. Introduction of leucoreduction occurred under the guidance of the technical team from the UBTS, with blood product inventory management procedures done for quality assurance in addition to residual leucocyte count. Laboratory and study staff did their study specific training by physical attendance. Acceptable online trainings were on good clinical practice (GCP) and good clinical laboratory practice (GCLP). Training sessions were tracked by the study coordinator and the PI to ensure consistency. Our study also endeavored to ensure patients were not lost to follow-up by phone calls to the participants and coordinating their return visits with the primary care team. The inclusion of patients with all grades of illness as measured by the ECOG physical performance scores implied that the study was pragmatic and therefore representative of the transfused patient population.

However, this study also had some limitations including overestimation of the effect of leucoreduced blood transfusion on mortality reduction as 15% during sample size estimation. The actual effect size based on the RCT was only 1.6%, implying that a larger sample size would have been required to demonstrate a mortality difference, if it existed between the two study arms. Though our retrospective study used to inform the mortality rate suggested a much higher rate [16] than what was observed in this study, incorporation of the mortality data in only patients with cancer in the retrospective study may have led to a more accurate sample size in retrospect. Additionally, monitoring the overall frequency of mortality early in the study would have identified the issue of low power and recruitment of patients with higher mortality could have been considered to resolve this issue. There might have also been a potential underreporting of the adverse events of transfusion by the adjudicating primary physicians (non-study personnel). This might have been mitigated by active follow up of the participants by the study team instead of relying on the documentation from routine care. Currently, there is no active haemovigilance system in Uganda; thus, transfusion reactions are assessed by the physicians based on their knowledge and experience. This contrasts with transfusion reaction adjudication using objective and published criteria, done by specialty transfusion medicine physicians and committees in some centres. Transfusion with other non-leucoreduced blood products including platelets were allowed in this study. Although this was pragmatic, the effect of transfusion with the non-leucoreduced blood products might have been a confounding variable. It might have been helpful to have these blood products leucoreduced, especially for the participants in the leucoreduced study arm.

## Conclusion

This study failed to demonstrate any mortality difference in patients with cancer transfused with leucoreduced or non-leucoreduced whole blood in a sub-Saharan African setting, though the pre-specified sample size was underpowered for the study given the observed mortality rate. As an exploratory outcome, there were no differences in acute blood transfusion reactions among the study participants, and increasing age and being less ill among study participants were associated with reduced mortality rate. Despite the lack of conclusive findings, this study illustrates that it is possible to undertake RCTs to evaluate different blood products in Uganda and to develop processes that support high quality methodological research. Such studies require careful consideration of the study population and other methodological considerations including sample size estimates.

## Data Availability

The datasets used and/or analysed during the current study is available from the corresponding author on reasonable request.

## Abbreviations

SSA: Sub-Sahara Africa
UCI: Uganda Cancer Institute
IQR: Interquartile Range
ECOG: Eastern Cooperative Oncology Group
RCT: Randomized Controlled Trial
CI: Confidence Interval
HR: Hazard Ratio
FNHTR: Febrile Non-haemolytic Transfusion Reaction
UBTS: Uganda Blood Transfusion Services
CPDA: Citrate Phosphate Dextrose Adenine
SOP: Standard Operating Procedures
GCP: Good Clinical Practice
GCLP: Good Clinical Laboratory Practice
DSMB: Data Safety and Monitoring Board
TRALI: Transfusion Associated Lung Injury
TTI: Transfusion Transmitted Infection
TACO: Transfusion Associated Circulatory Overload
TA-GVHD: Transfusion Associated Graft Versus Host Disease
SD: Standard Deviation
Hb: Haemoglobin
